# Critical role of STAT3 in melanoma metastasis through anoikis resistance

**DOI:** 10.18632/oncotarget.2251

**Published:** 2014-07-25

**Authors:** Neel M. Fofaria, Sanjay K. Srivastava

**Affiliations:** ^1^ Department of Biomedical Sciences & Cancer Biology Center, Texas Tech University Health Sciences Center, Amarillo, Texas, USA

**Keywords:** STAT3, anoikis resistance, metastasis, melanoma

## Abstract

Anoikis is an anchorage-independent cell death. Resistance to anoikis is one of the key features of metastatic cells. Here, we analyzed the role of STAT3 in anoikis resistance in melanoma cells leading to metastasis. When grown under anchorage-independent conditions, significant proportion of cells resisted anoikis and these resistant cells had higher rate of migration and invasion as compared to the cells grown under anchorage-dependent conditions. The anoikis resistant cells also had significantly higher expression and phosphorylation of STAT3 at Y705 than the cells that were attached to the basement membrane. STAT3 inhibitors, AG 490 and piplartine (PL) induced anoikis in a concentration-dependent manner in anoikis resistant cells. Over-expression of STAT3 or treatment with IL-6 not only increased anoikis resistance, but also protected the cancer cells from PL-induced anoikis. On the other hand, silencing STAT3 decreased the potential of cancer cells to resist anoikis and to migrate. STAT3 knock-down cells and PL treated cells did not form tumors as well as failed to metastasize in SCID-NSG mice as compared to untreated anchorage-independent cells, which formed big tumors and extensively metastasized. In summary, our results for the first time establish STAT3 as a critical player that renders anoikis resistance to melanoma cells and enhance their metastatic potential.

## INTRODUCTION

Cells grow and differentiate when they are in contact with the extracellular matrix (ECM)[1]. Cells deprived of attachment to ECM undergo classical apoptosis known as anoikis, meaning ‘homelessness’ in Greek[2]. Epithelial cells highly depend on appropriate cell-cell and cell-matrix environment for survival[3]. However, tumor cells that acquire malignant potential develop ways to resist anoikis and survive under anchorage-independent conditions and acquire migratory potential[4]. Hence, tumor cells need to evade anoikis in order to metastasize, making anoikis resistance a key feature of metastatic cancers. Anoikis is a new yet critical concept that is recently being the focus of scientific attention. However, the exact mechanism of anoikis resistance is yet not known[5].

Signal Transducers and Activators of Transcription (STAT) family of transcription factors play a critical role in the expression of genes that are involved in cell differentiation, survival, proliferation, chemoresistance and angiogenesis[5-13]. Enhanced STAT3 activity has been observed in various types of human cancers[14-18]. Janus-activated kinases (JAK), Interleukin-6 (IL-6), epidermal growth factor receptors and Src kinases activate STAT3 by phosphorylation at important tyrosine residues. Phosphorylation of STAT3 causes dimerization of STAT3 followed by its nuclear translocation where it enhances the transcription of target genes[13, 19-21]. One of the most critical sites of phosphorylation is at Tyrosine 705 (Y705), which enhances the expression of various proliferation and survival genes such as Bcl-2, Mcl-1, Cyclin D1 and survivin[14, 22, 23].

Melanoma is a neoplasm of melanocytes which accounts for highest number of skin cancer related deaths. In 2014, 76,100 new cases will be diagnosed out of which 9,710 people are expected to die (http://www.cancer.org/cancer/skincancer-melanoma/detailedguide/melanoma-skin-cancer-key-statistics). The 5-year survival probability of the patient suffering from melanoma is less than 5% and once it metastasizes, the probability of survival goes to less than 1%. [24]. Dacarbazine, which was approved three decades ago, still remains a drug of choice to treat malignant melanoma although it benefits only a small subset of patients [25]. Most common sites of metastases for melanoma are lymph nodes, lungs, liver, bones and brain. Melanoma cells which are resistant to anoikis can easily metastasize [4] thus, making melanoma an important tumor model to study anoikis resistance [26, 27].

In the current study, we have established a critical role of STAT3 in anoikis resistance *in vitro* and *in vivo* in melanoma. Furthermore, our study demonstrates that induction of anoikis resistance was associated with enhanced cell migration, invasion and metastasis in various *in vivo* tumor models. To the best of our knowledge, this is the first study establishing a direct role of STAT3 in anoikis resistance in melanoma.

## RESULTS

### Melanoma cells resist anoikis in anchorage-free conditions

**Figure 1:**
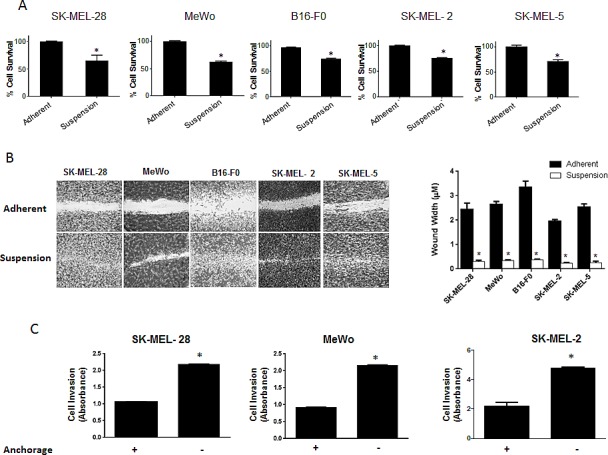
Significant population of melanoma cells resist anoikis in anchorage independent conditions (A) SK-MEL-28, MeWo, B16-F0, SK-MEL-2 and SK-MEL-5 cells were cultured under anchorage independent conditions in the plates coated with poly-HEMA for 48 hours and then replated in 24-well plate. The cells were then allowed to attach after which the cell viability was evaluated using Sulforhodamine B assay. The cell survival was compared with the cells cultured under adherent conditions for same time period. Anoikis resistant cells are highly migratory and invasive. (B) Human melanoma cells SK-MEL-28, MeWo, SK-MEL-2, SK-MEL-5 and murine melanoma cells B16-F0 were cultured under adherent or suspension conditions for 48 hours and then replated in a 24-well plate. Confluent monolayers were scratched with 1 mL pipette tip. Wounds were allowed to heal for 16 hours and imaged by microscope. (C) Invasion of SK-MEL-28, MeWo and SK-MEL-2 cells was measured by Boyden's Transwell assay according to the manufacturer's instructions. Values are plotted as mean ± S.D. *, p < 0.05 compared with adherent group. Each experiment was repeated at least three times with similar results.

Anoikis is a form of cell death that occurs when the cells detach from the basement membrane. Studies in the past have shown that cancer cells are able to resist anoikis and hence, they metastasize (4). However, the exact molecular mechanism why few cells resist anoikis and acquire metastatic potential is not known. Using anoikis assay, we screened five melanoma cell lines for their potential to resist anoikis. All the five cell lines used were malignant melanoma cell lines and were isolated from metastatic sites. SK-MEL-28, SK-MEL-2, SK-MEL-5, MeWo and B16-F0 cells were cultured under low attachment (anchorage-free) conditions for 48 hours after which their survival was evaluated by the Sulforhodamine B (SRB) assay and compared with the cells under adherent conditions for the same time period. Notable anoikis was induced in all the cancer cell lines when cultured under anchorage-free conditions (Fig. 1A). More importantly, a significant percentage of cells survived and were termed as anoikis resistant cells. In SK-MEL-28 and MeWo, about 65% of cells resisted anoikis and in SK-MEL-2, SK-MEL-5 and B16 –F0, about 75% of cells resisted anoikis when cultured under anchorage independent conditions (Fig. 1A)

### Anoikis resistant cells are highly migratory and invasive

Recent studies have shown that it is only after the cancer cells resist anoikis that they attain the potential to metastasize[4]. Migration and invasion are one of the most critical steps in metastasis as the cells in the circulation need to migrate and invade the secondary organs. Hence, we performed migration and invasion assays using anoikis resistant cells. Cells were incubated either in suspension or adherent conditions for 48h and transferred to 24 well plates. A wound healing assay was performed in five melanoma cell lines. The experiment was terminated within 16 hours after creating the wound. Our results showed that cells that were cultured under anchorage-independent conditions and evaded anoikis, healed the wound at much higher rate than adherent cells (Fig. 1B). Furthermore, invasion assay using Boyden's chamber was performed in SK-MEL-28, SK-MEL-2 and MeWo cells. Our results showed that anoikis resistant cells were highly invasive as compared to adherent cells (Fig. 1C). SK-MEL-28 and MeWo exhibited 2 fold higher rate of invasion and SK-MEL-2 cells showed 2.5 fold higher rate of invasion as compared to their respective adherent controls (Fig 1C). Hence, these results indicate that the cells that resisted anoikis were highly migratory and invasive.

### STAT3 is overexpressed in anoikis resistant melanoma cells

**Figure 2:**
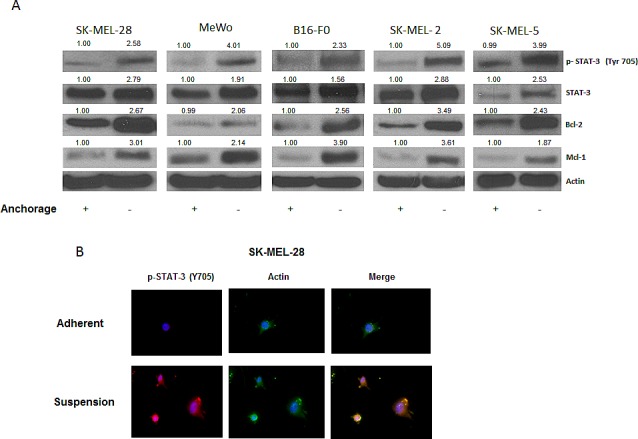
STAT3 is overexpressed in anoikis resistant cells (A) Representative blots of pSTAT3, STAT3, Bcl-2 and Mcl-1 from lysates of human melanoma cells SK-MEL-28, MeWo, B16-F0, SK-MEL-2, and SK-MEL-5 cultured under anchorage dependent or independent culture conditions for 48 hours. Lysates were prepared and the protein was subjected to western blotting. Actin was used as loading control. (B) SK-MEL-28 cells cultured under adherent or suspension culture conditions were immunostained for pSTAT3 (Y705) (red) or actin (green). Nucleus was stained using DAPI. Each experiment was repeated at least three times with similar results.

Our results showed that anoikis resistant cells had a very high potential to migrate and invade, as compared to the adherent cells. The next step was to find out what molecular changes occured in these cells making them resistant to anoikis and transformed them into such highly migratory and invasive phenotype. To investigate this, we evaluated the expression of various proteins in the anoikis resistant melanoma cells (SK-MEL-28, SK-MEL-5, SK-MEL-2, MeWo and B16-F0) and compared the results with respective adherent controls. As compared to adherent cells, a remarkable phophorylation of STAT3 at Y705 was observed in all the five melanoma cell lines cultured under anchorage-independent conditions and resisted anoikis (Fig. 2A). There was also a significant up-regulation of STAT3 protein expression in anoikis resistant cells (Fig. 2A). Furthermore, anoikis resistant cells showed marked increase in the expression of anti-apoptotic proteins Bcl-2 and Mcl-1 that are under the transcriptional regulation of STAT3 (Fig 2A). These observations were also confirmed by immunofluorescence. As compared to adherent cells, substantial staining of p-STAT3 (Y705), which was indicated by red fluorescence, was observed in SK-MEL-28 cells that were cultured under anchorage-independent conditions (Fig. 2B). Actin was indicated by green stain. Based on these results, we hypothesized that STAT3 plays a critical role in imparting anoikis resistance to cancer cells and promotes their migration and invasion potential leading to metastasis

### AG 490 and Piplartine suppress resistance to anokis in cancer cells

**Figure 3:**
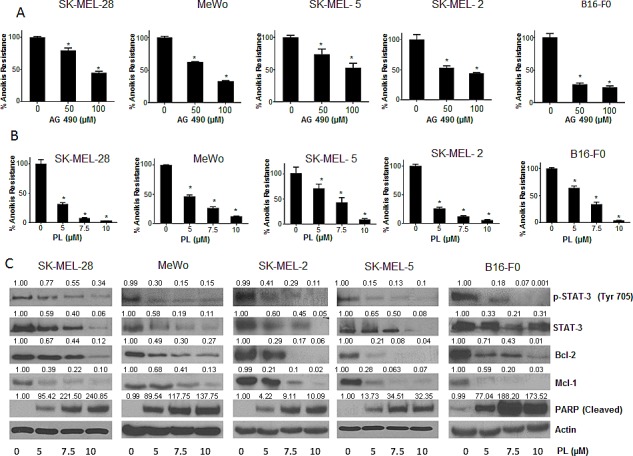
STAT3 inhibitors induce anoikis in cancer cells (A) Human melanoma cells SK-MEL-28, MeWo, SK-MEL-5, SK-MEL-2 and murine melanoma cells B16-F0 were cultured in plates coated with poly-HEMA as suspension culture (anchorage independent condition) and treated with DMSO or various concentrations of AG 490. After 48 hours, cells were replated in 24 well plated and the viable cells were analyzed by Sulforhodamine B assay. Representative bar graphs show the percentage anoikis resistance in different treatment conditions. Values are plotted as mean ± S.D. *, p < 0.05 compared with control group. (B) Human melanoma cells SK-MEL-28, MeWo, SK-MEL-5, SK-MEL-2, B16-F0 were cultured in plates coated with poly-HEMA under anchorage independent condition and treated with DMSO or various concentrations of piplartine (PL). After 48 hours, cells were replated in 24 well plate and the viable cells were analyzed by Sulforhodamine B assay. Representative bar graphs show the percentage anoikis resistance in different treatment conditions. Values are plotted as mean ± S.D. *, p < 0.05 compared with control group. PL reverses anoikis resistance by inhibition of STAT3. (C) Blots are representative of pSTAT3 (Y705), STAT3, Bcl-2, Mcl-1 and cleaved PARP from lysates collected from human melanoma cells SK-MEL-28, MeWo, SK-MEL-2, SK-MEL-5 and B16-F0 grown under anchorage independent conditions and treated with DMSO or various concentrations of PL for 48 hours. Actin was used as loading control. Each experiment was repeated at least three times with similar results.

To test our hypothesis that STAT3 plays a role in anoikis resistance, we wanted to determine whether STAT3 inhibitors can overcome aniokis resistance in cancer cells. For this purpose, we used a known synthetic STAT3 inhibitor AG 490. Five melanoma cell lines (SK-MEL-28, SK-MEL-2, SK-MEL-5, MeWo and B16-F0) were treated with 50μM or 100μM AG 490 under anchorage independent conditions for 48 hours, after which the cells were re-cultured on an adherent plate where only the anoikis resistant cells attached and survived. The survival of the treated cells was evaluated using the SRB assay and compared with the untreated anoikis resistant cells. Our results demonstrated that AG 490 was able to substantially reduce anoikis resistance in all the melanoma cell lines (Fig. 3A). Anoikis resistance by treatment with 50-100μM AG 490 was reduced by 20-60% in SK-MEL-28 cells (Fig. 3A). Similar concentrations of AG 490 reduced anoikis resistance by 45-65% in SK-MEL-2, 25-50% in SK-MEL-5, 30-65% in MeWo and 70-75% in B16-F0 cells (Fig. 3A).

We also evaluated the effects of piplartine (PL), a component of black pepper, on anoikis resistance of cancer cells as PL was found to suppress the growth of various cancer cell lines in our previous unpublished observations. Our current results showed that PL (5-10 μM) after 48 h treatment caused a significant concentration-dependent reduction of anoikis resistance in all the melanoma cells (Fig. 3B). PL at 10 μM reduced the anoikis resistance by 90 % in most of the cell lines tested (Fig. 3B).

### PL reverses anoikis resistance by inhibiting STAT3

Since PL was superior to AG 490 in suppressing anoikis resistance in all the cancer cell lines tested, we wanted to identify the mechanism of anoikis induction by PL. Five melanoma cell lines (SK-MEL-28, SK-MEL-2, SK-MEL-5, MeWo and B16-F0) were cultured under anchorage- independent conditions, treated with various concentrations of PL for 48 hours and analyzed by western blotting. Our results showed that PL treatment significantly decreased the phophorylation of STAT3 at Y705 in a concentration-dependent manner in all the melanoma cell lines (Fig. 3C). At the highest concentration, PL suppressed almost 90% phophorylation of STAT3 (Fig. 3C). Furthermore, PL also down-regulated the protein levels of STAT3 in all the cell lines tested (Fig. 3C). Marked down-regulation of Mcl-1 and Bcl-2, the anti-apoptotic proteins, which are under transcriptional regulation of STAT3, were also observed by PL treatment (Fig. 3C). Massive cleavage of PARP indicated induction of anoikis by PL treatment (Fig. 3C). These results clearly indicated that STAT3 inhibitors significantly reduce anoikis resistance in melanoma cells.

### Silencing STAT3 using shRNA blocks anoikis resistance in cancer cells

**Figure 4:**
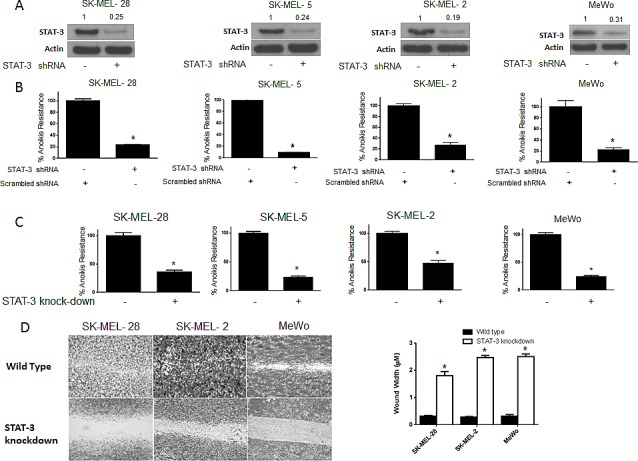
STAT3 deficient cells are sensitive to anoikis and lose migratory potential (A-B) SK-MEL-28, SK-MEL-5, SK-MEL-2 and MeWo cells were transfected with STAT3 shRNA for 24 hours after which they were cultured under anchorage independent condition for 48 hours. Cells transfected with scrambled shRNA and cultured under similar conditions were used as control. Percentage of STAT3 silencing was tested by western blotting. Values are plotted as mean ± S.D. *, p < 0.05 compared with control group. Extent of silencing was evaluated by western blotting prior to the experiment and shown by the representative blots for every cell line. (C) STAT3 knock-down human melanoma cell lines SK-MEL-28, SK-MEL-2, SK-MEL5 and MeWo were cultured in anchorage-independent conditions for 48 hours. Cells were replated in a 24-well plate and the viable cells were measured by SRB assay. Wild-type cells of the respective cell lines were used as control. Values are plotted as mean ± S.D. *, p < 0.05 compared with control group. (D) STAT3 knock-down human melanoma cell lines SK-MEL-28, SK-MEL-2 and MeWo cells were cultured in plates coated with poly HEMA. After 48 hours, cells were transferred to a 24-well plate and wound healing assay was performed as described earlier. Respective wild-type cells cultured under similar conditions were used as control. Each experiment was repeated at least three times with similar results

To further test our hypothesis and to confirm the role of STAT3 in anoikis resistance, we silenced STAT3 using shRNA in four human melanoma cells lines (SK-MEL-28, SK-MEL-2, SK-MEL-5 and MeWo). About 80-90% silencing of STAT3 was achieved using shRNA in all the four cell lines (Fig. 4A). Anoikis assay was performed in these STAT3 silenced cells using the wild type cells transfected with scrambled shRNA as controls. Our results demonstrated that silencing of STAT3 highly sensitized the cells to anoikis (Fig. 4B). Inhibition of STAT3 caused about 80-90% decrease in anoikis resistance in all of the cell lines (Fig. 4B), which highly correlated with STAT3 silencing. The percentage of STAT3 silencing was similar in all the cell lines and so was the reduction in anoikis resistance (Fig. 4A-B). These results confirmed the role of STAT3 in providing anoikis resistance to melanoma cells.

### STAT3 knock-down cells are more sensitive to anoikis and lose migratory potential

STAT3 knock-down cell lines were used to further confirm the role of STAT3 in anoikis. We developed stable STAT3 knock-down (KO) melanoma cells for this purpose. We stably silenced STAT3 in SK-MEL-28, SK-MEL2, SK-MEL-5 and MeWo cells. Both STAT3 (WT) and STAT3 knock-down (KO) cells were grown under similar anchorage-independent conditions for 48 hours following which anoikis resistance was determined in these cells. As expected, anoikis resistance was decreased by 60-75% in STAT3 knock-down as compared to the wild type counterparts in all four cell lines tested (Fig. 4C). Since our results showed that anoikis resistance was associated with enhanced migratory potential, we performed a wound-healing assay in three STAT3 knock-down and respective STAT3 (WT) melanoma cells. We observed that STAT3 knock-down cells had marked reduction in the wound healing process as compared to STAT3 (WT) cells (Fig. 4D). In fact, STAT3 knock-down cells completely failed to heal the wound by 16 hours as compared to STAT3 wild type anoikis resistant cells (Fig. 4D). Hence, knocking out STAT3 not only decreased anoikis resistance but also inhibited the migratory potential of anoikis resistant cancer cells (Fig. 4D). Taken together, these results establish the role of STAT3 in anoikis resistance and migration in melanoma cells.

### IL-6 enhances anoikis resistance in cancer cells and blocks PL-induced anoikis

**Figure 5:**
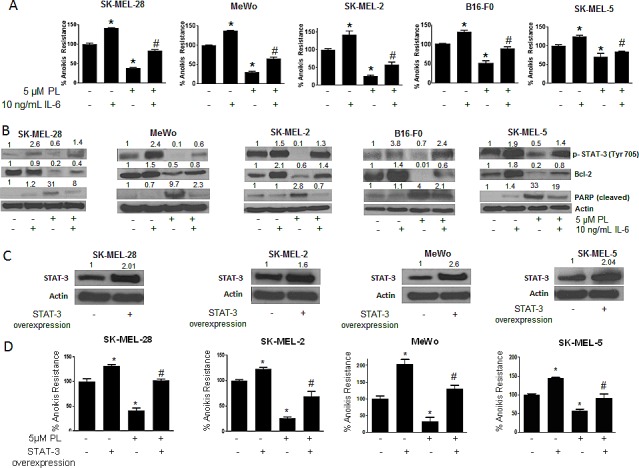
IL-6 and STAT3 over-expression enhances anoikis resistance in cancer cells and reverses anoikis sensitization by PL (A) SK-MEL-28, MeWo, SK-MEL-2, B16-F0 and SK-MEL-5 cells were treated with IL-6 alone or in combination with 5 μM PL under anchorage independent condition. After 48 hours, cells were transferred to 24-well plate and the viability was measured by Sulforhodamine B assay. Representative bar graph shows percent anoikis resistance of cells exposed to various treatments under anchorage independent conditions. Values are plotted as mean ± S.D. *, p < 0.05 compared with control group and #, p < 0.05 when compared with PL treated cells. (B) Representative blots of pSTAT3, Bcl-2 and Cleaved PARP from of lysates of SK-MEL-28, MeWo, SK-MEL-2, B16-F0 and SK-MEL-5 cells treated with IL-6 alone or in combination with 5 μM PL for 48 hours. Actin was used as a loading control. (C-D) SK-MEL-28, SK-MEL-2, MeWo and SK-MEL-5 cells were transfected with STAT3 overexpressing plasmid. After 24 hours, cells were transferred to poly HEMA coated plates treated with or without 5 μM PL. After 48 hours, cells were replated in a 24-well plate and the viable cells were analyzed by SRB assay. Representative bar graph shows percent anoikis resistance after various treatments. Values were plotted as mean ± S.D. *, p < 0.05 compared with control cells and #, p < 0.05 when compared with PL treated cells. Fold increase in overexpression was evaluated by western blotting prior to the experiment and a representative blot is shown in the figure. Each experiment was repeated at least three times with similar results

To further strengthen the role of STAT3 in anoikis resistance, IL-6 was used to activate STAT3. IL-6 is well known to activate STAT3 by phosphorylation at Y705[28]. As we had hypothesized that phosphorylation at of STAT3 Y705 conferred anoikis resistance to melanoma cells, IL-6 treatment would enhance anoikis resistance in these cells. Moreover, since our results indicated that PL induced anoikis by inhibition of p-STAT3 (Y705), we hypothesized that IL-6 treatment would block PL-induced anoikis. To test the effect of IL-6, melanoma cells were pre-treated with IL-6 and then treated with PL under anchorage-independent conditions for 48 hours. As shown in Fig. 5A, IL-6 treatment significantly increased anoikis resistance in all the melanoma cell lines as compared to untreated controls. IL-6 treatment increased the anoikis resistance by 1.5 fold in SK-MEL-28 and SK-MEL-2, by 1.4 fold in MeWo cells and by 1.3 fold in SK-MEL-5 and B16-F0 cells (Fig. 5A). As expected, IL-6 also provided marked protection to PL treated cells (Fig. 5A). IL-6 treatment significantly blocked the reduction in anoikis resistance mediated by PL treatment in all five cell lines (Fig. 5A). For example, in SK-MEL-28 cells, PL reduced anoikis resistance by 65% (Fig. 5A). However, IL-6 treatment blocked PL mediated anoikis by almost 45% (Fig. 5A). Similarly, significant reduction in anoikis resistance by PL treatment in SK-MEL-2, SK-MEL-5, MeWo and B16-F0 cells was blocked by IL-6 treatment, confirming our hypothesis (Fig. 5A).

To further support these observations, our western blot results showed a massive increase in the phosphorylation of STAT3 at Y705 upon IL-6 treatment (Fig. 5B). Our results also revealed that reduction of p-STAT3 (Y705) by PL treatment was significantly blocked by IL-6 (Fig. 5B). IL-6 also blocked PL-mediated inhibition of the expression of Bcl-2 (Fig. 5B). Our results also showed that IL-6 treatment decreased the cleavage of PARP that was induced by PL treatment in all the cell lines (Fig. 5B). These results validated the role of STAT3 in rendering anoikis resistance to melanoma cells.

### STAT3 over-expression augment anoikis resistance in cancer cells

Since our results indicated the involvement of STAT3 in anoikis resistance, we finally wanted to see if STAT3 over-expression could enhance anoikis resistance. We over-expressed STAT3 by transfecting STAT3 expressing plasmid in four melanoma cell lines (SK-MEL-28, SK-MEL-2, SK-MEL5 and MeWo) and performed anoikis assay. We were able to achieve 1.6-2.6 fold increased expression of STAT3 (Fig. 5C). Anoikis resistance was substantially increased in all the cells over-expressing STAT3 (Fig. 5D). The increase in anoikis resistance due to STAT3 over-expression was 1.3 fold in SK-MEL-28 and SK-MEL-2 cells, 1.5 fold in SK-MEL-5 cells and 2 fold in MeWo cells (Fig. 5D). The increase in anoikis resistance highly correlated with the level of STAT3 overexpression. For example maximum overexpression was achieved in MeWo cells which in turn had the highest increase in anoikis resistance (Fig. 5C-D). Because our results showed that PL treatment reduced anoikis resistance by inhibiting STAT3, we wanted to see if STAT3 over-expression could block the effects of PL. Our results revealed that PL mediated anoikis was substantially abrogated in STAT3 over-expressing cells (Fig. 5D). For example, in SK-MEL-28 cells, PL reduced anoikis resistance by 65% (Fig. 5D). Upon STAT3 overexpression, the induction of anoikis was completely nullyfied (Fig. 5D). Similarly, STAT3 overexpression significantly abrogated PL-induced anoikis in SK-MEL-2, SK-MEL-5 and MeWo cells. Taken together, these results once again conclusively established the role of STAT3 in anoikis resistance in melanoma.

### STAT3 deficient cells failed to form tumors *in vivo*

**Figure 6:**
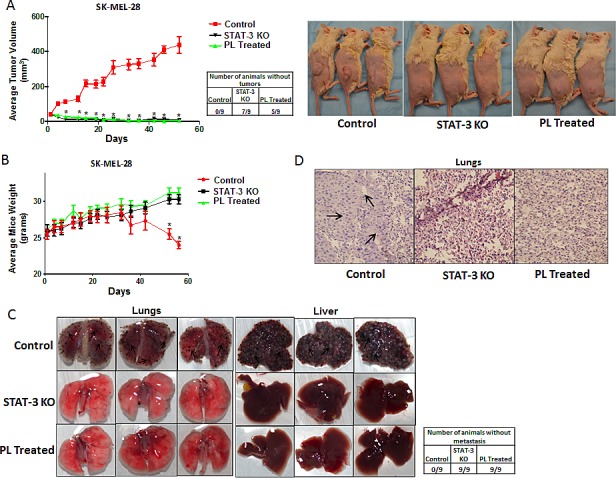
STAT3 deficient cells failed to form tumor *in vivo* (A) Tumor volumes and images of the mice bearing xenografts of wild type (Control), STAT3 knockout (STAT3 KO) and PL treated (5μM) SK-MEL 28 cells cultured under anchorage independent conditions for 48 hours. Values are plotted as mean ± S.E.M. *, p < 0.05 compared with control group. Cell viability of each group was evaluated using trypan blue assay and 5 × 10^6^ live cells were injected. Once palpable tumors were observed, tumor dimensions were measured using vernier calipers. Values are plotted as mean ± S.E.M. *, p < 0.05 compared with control group. STAT3 enhances metastatic potential in melanoma cells by rendering anoikis resistance. (B) Animal weight (C) Images of lungs and liver and (D) Hematoxylin and Eosin staining of lung sections of the mice bearing metastatic nodules of wild type (Control), STAT3 knock-out (STAT3 KO) and PL treated (5μM) SK-MEL- 28 cells cultured under anchorage independent conditions for 48 hours and injected intravenously. Live cells were counted by trypan blue staining and 0.2 × 10^6^ live cells were injected intravenously. The experiment was continued till the mice from any of the group started dying due to metastatic burden. Arrows indicate presence of metastatic nodules.

In order to validate the critical role of STAT3 in anoikis resistance in the *in vivo* model, melanoma tumor xenograft experiments were performed in SCID-NSG mice. Three different groups of mice (n=9/group) were taken. About 5 × 10^6^ live anchorage-independent cells were injected subcutaneously in both the flanks of SCID-NSG mice to determine the tumor formation ability. Mice in group 1 were implanted with SK-MEL-28 wild type anoikis resistant cells (Control), group two mice received SK-MEL-28 STAT3 knockout (STAT3 KO) and group three mice received SK-MEL-28 anoikis resistant cells treated with 5 μM PL for 48 h. Once palpable tumors were formed, tumor measurements were taken twice a week using vernier calipers. Our results showed that SK-MEL-28 wild type cells formed very aggressive tumors with an average tumor volume of 440 ± 47.86 mm^3^ by the end of day 50 (Fig. 6A). However, STAT3 knockout or PL treated SK-MEL-28 cells completely failed to form tumors (Fig. 6A). The average tumor volume in mice from STAT3 KO group was 9 ± 2.9 mm^3^ and that in mice from PL treated group was 6 ± 2 mm^3^ (Fig. 6A). Seven mice in KO group and 5 mice in PL group were completely free of tumors (Fig. 6A). These results indicated that cells with reduced or no expression of STAT3 were highly sensitive to anoikis and, therefore, failed to grow as tumors. On the other hand, wild type cells where STAT3 was activated during anchorage-independent conditions had very high tumorigenic potential. Fig. 6A also shows the images of tumor bearing mice from the three groups. As compared to control mice, STAT3 KO mice had no palpable tumors whereas PL treated mice showed very small size tumors which failed to grow over the duration of the experiment (Fig. 6A).

### STAT3 enhances metastatic potential in cancer cells by rendering anoikis resistance

Anoikis resistance is one of the hallmarks of a metastatic cancer cell. As a proof-of-principle, we tested the role of STAT3 in the metastasis of melanoma in the animal model. Anchorage-independent SK-MEL-28 wild type (Control), SK-MEL-28 STAT3 knockout (STAT3 KO) or PL treated SK-MEL-28 cells were injected intravenously in mice after which their ability to metastasize was determined. The weight of the mice injected with SK-MEL-28 STAT3 knockout or PL treated SK-MEL-28 cells increased normally over the period of time (Fig. 6B). However, a significant reduction in the weight of the mice injected with SK-MEL-28 wild type cells was observed (Fig. 6B). The weight reduction of mice in this group indicated illness due to the heavy metastatic burden. This is the time when the animals were sacrificed and the extent of metastasis was examined. Our results showed that mice injected with anoikis resistant SK-MEL-28 wild type cells had an extensive metastatic burden. The cells mainly metastasized to lungs and liver (Fig. 6C). Lungs and livers of mice from anoikis resistant wild type group showed massive metastatic nodules. Being melanotic, SK-MEL-28 cells appeared as pigmented nodules (Fig. 6C) [29-35]. Surprisingly, no observable nodules were found in the lungs and livers of the mice injected with STAT3 knockout (STAT3 KO) or PL treated cells, which supposedly had diminished expression of STAT3 (Fig. 6C). H & E staining was performed to confirm the presence of metastatic nodules. The metastatic nodules in the lung sections were depicted by the presence of large and randomly arranged nuclei, a typical characteristic of a tumor (Fig. 6D). As expected, no metastatic nodules were detected in the sections of the lungs of STAT3 knockout or PL treated groups (Fig. 6D). Taken together, these results demonstrated that STAT3 plays a very important role in metastasis of melanoma tumors *in vivo*.

## DISCUSSION

In the current study, we established the role of STAT3 in anoikis resistance and metastasis in melanoma *in vitro* and *in vivo*. The key findings of this study are that STAT3 plays a very important role in anoikis resistance and inhibiting STAT3 induces anoikis in cancer cells *in vitro* and *in vivo*. Moreover, induction of anoikis resistance by STAT3 was also associated with enhanced cell migration and invasion of cancer cells *in vitro* and high metastatic potential *in vivo*.

Our results showed that all the melanoma cell lines were highly resistant to anoikis and only a small percentage of cells were sensitive to anoikis. Based on the fact that the cancer cells that resist anoikis will eventually metastasize, it is very likely that these cells would also have very high potential to migrate and invade, to facilitate their seeding and invading to distant organs. In a wound healing assay and Boyden's chamber assay our data suggests enhanced migratory potential and invasiveness of anoikis resistant cells as compared to adherent cells in various cell lines tested.

STAT3 is involved in diverse functions including cell growth, survival, differentiation, inflammation, immune system and apoptosis. There have been few reports showing the role of STAT3 in anoikis in squamous cell carcinoma and hepatocellular carcinoma [36-38]. However, none of these studies demonstated any *in vivo* evidence indicating the role of STAT3 in anoikis resistance or metastasis. Nonetheless, the role of STAT3 in anoikis and metastasis in melanoma has not been established yet. As compared to adherent cells, anoikis resistant melanoma cells exhibited significantly increased expression and phosphorylation of STAT3 at Y705. Our results demonstrated increased expression of Bcl-2 and Mcl-1 in anoikis resistant cells associating STAT3 with anoikis resistance. Based on these results, we hypothesized that activation and over-expression of STAT3 induces anoikis resistance in cancer cells and promotes metastasis.

Our results demonstrated almost 70-80% reduction in anoikis resistance upon transient silencing of STAT3. Interestingly, anoikis induction in these cells highly correlated with the extent of STAT3 knockdown. The extent of knockdown was comparable in all the melanoma cell lines and consequently the reduction in anoikis resistance was also similar indicating that all the metastatic melanoma cell lines were equally dependent on STAT3 for anoikis resistance. These results were also confirmed in STAT3 knock-down melanoma cell lines. The role of STAT3 in anoikis resistance has also been confirmed in pancreatic cancer (unpublished data). STAT3 knock-down cells not only had lower resistance to anoikis, but also exhibited a lower rate of migration as compared to their respective wild type anchorage-independent cells. On the other hand, IL-6 and over-expression of STAT3 in SK-MEL-28, SK-MEL-2, SK-MEL-5 and MeWo cells not only decreased sensitivity to anoikis but also completely protected from PL-induced anoikis. A significant population of cells that was sensitive to anoikis became resistant upon enhancing the expression of STAT3 or its phosphorylation at Y705. Induction of anoikis by PL mediated inhibition of STAT3 has not been reported. Our results showed that PL was more potent than AG 490 in inducing anoikis. Although AG 490 is a STAT3 inhibitor, there have been several reports indicating its off-target effects, for e.g. AG 490 has been shown to inhibit EGFR as well as HER-2[39]. In addition, it has also been shown to inhibit Akt[40]. This could be a reason for its decreased potency. Abrogation of PL-induced anoikis by STAT3 overexpression or IL-6 treatment confirmed the role of STAT3 in PL induced anoikis. Taken together, these results clearly showed that STAT3 plays a critical role in conferring anoikis resistance and promoting cell migration in cancer cells. The cells lines used for this study had differences in their genetic profile. For instance, SK-MEL-28 and SK-MEL-5 had mutant BRAF^V600E^ whereas the other cell lines used had wild-type BRAF. SK-MEL-2 and SK-MEL-5 had mutant N-RAS whereas all the others had wild type NRAS. SK-MEL-28 and MeWo harbored mutant p53, whereas the other cells lines had wild type p53[41, 42]. In spite of these genetic variations in these cell lines, all of these cell lines demonstrated similar dependence on STAT3 for anoikis resistance.

Importantly, SK-MEL-28 exposed to PL under anchorage-independent conditions completely failed to grow as tumors when injected subcutaneously in SCID/NSG mice. Similarly, STAT3 knock-down SK-MEL-28 cells cultured under anchorage-independent conditions also failed to form tumors *in vivo*. On the contrary, wild type untreated SK-MEL-28 cells cultured under anchorage-independent condition formed aggressive tumors in SCID/NSG mice. Failure to form tumors by PL treatment or STAT3 knock-down cells can be attributed to the diminished expression of STAT3, which consequently led to increase sensitivity to anoikis.

It is noteworthy that PL treated and STAT3 knock-down anchorage-independent SK-MEL-28 cells completely failed to metastasize to lungs and liver when injected intravenously in SCID-NSG mice. However, SK-MEL-28 wild type cells cultured under similar conditions extensively metastasized to lungs and liver upon intravenous injection. These observations once again show that the cancer cells with differential expression of STAT3 respond differently to anoikis resistance and metastasis. Our study also supports the accumulating clinical and pathological evidence on the role of STAT3 in the aggressiveness of various cancers[14, 15]. Moreover, the role of STAT3 in anoikis resistance and metastasis *in vivo* has not been established yet.

In conclusion, we report a novel characteristic of STAT3 in promoting resistance to anoikis in melanoma. These results strongly establish overexpression of STAT3 as a critical mechanism to evade anoikis in melanoma and promote metastasis. Therefore, STAT3 inhibitors can be used as a rational therapeutic strategy to prevent or inhibit metastasis in melanoma.

## MATERIALS AND METHODS

### Chemicals

Piplartine (PL) was obtained from Cayman Chemicals (Ann Arbor, MI). AG 490 was acquired from Selleck Chemicals (Houston, TX). G418, Mayer's Hematoxylin, Eosin and Permount were obtained from Fisher Scientific (Houston, TX). All the antibodies were bought from Cell Signaling (Danvers, MA) unless specified. Poly(2-hydroxyethyl) methacrylate (poly-HEMA), Sulforhodamine (SRB) and antibody against actin were obtained from Sigma-Aldrich (St. Louis, MO). RPMI and McCoy 5A were purchased from Mediatech (Manassas, VA). DMEM and EMEM were procured from ATCC (Manassas, VA). Transfection reagent Lipofectamine 2000 was obtained from Life Technologies (Grand Island, NY). Recombinant IL-6 was purchased from Peprotech (Rockyhill, NJ). STAT3 shRNA was obtained from SA Biosciences (Frederick, MD) and STAT3α plasmid was a generous gift from Dr. J.F. Bloomberg (Rockefeller University, NY).

### Cell Culture

Human melanoma cell lines SK-MEL-28, MeWo, and murine melanoma cell line B16-F0 were obtained from ATCC (Manassas, VA) and cultured as previously described [43]. Human melanoma cell line SK-MEL-2 was a kind gift from Dr. Srikumar Chellappan (H. Lee Moffitt Cancer Center and Research Institute, Tampa, FL) and SK-MEL-5 was a gift from Dr. Randy Burd (University of Arizona, Tucson, AZ). All the melanoma cell lines were cultured in EMEM medium (ATCC, Manassas, VA) supplemented with 5% fetal bovine serum and antibiotics (penicillin, streptomycin, neomycin). All the cell lines were authenticated by short tandem repeat (STR) analysis at Texas Tech University Health Sciences Center (TTUHSC) core facilities (Lubbock, TX).

### Anoikis assay

In order to simulate anchorage-independent growth conditions, we coated culture dishes with Poly-HEMA as described earlier[44]. Anoikis assay was performed according to the previously described protocol by us[44]. Approximately 1×10^6^ cells were plated in poly-HEMA coated petri dishes. These cells were either untreated or treated with various concentrations of piplartine (PL) or AG 490. After the desired treatment time was achieved, cells were centrifuged and uniformly divided in a 24-well plate. As previously described, after 8 hours, cells were processed for SRB assay[45]. Anoikis resistance of control was considered as 100% and various treatments were calculated as percentage of control. The graph was then plotted as percent of anoikis resistance on the y-axis and various treatment concentrations on x-axis.

### Migration and Invasion Assay

Wound Healing and invasion assays were performed to compare the migratory potential and invasiveness of anoikis resistant cells and compared with the adherent cells. This assay was performed according to the previously described method by us[46-48]. Cells were incubated in either anchorage dependent or independent conditions for 48. For wound healing assay, cells were transferred to a 24-well plate. Eight hours after the formation of the monolayer, a wound was made across it by scratching with 1mL sterile pipette tip. Cells were washed to remove displaced and floating cells and allowed to migrate until one of the groups healed the wound completely (usually 16 hours), followed by staining with SRB. The cells were imaged under microscope. (Olympus America, Inc, Center Valley, PA)

For invasion assay, after 48 hours incubation of cells under adherent or anchorage independent conditions, cells were transferred to the upper chamber of Boyden's chamber (BD Biosciences, Bedford, MA) and were serum starved for 24 hours after which VEGF (10 ng/mL) was added to the lower chamber as a chemo attractant. After 48 hours, cells in the upper chamber were wiped off with a cotton swab and the filter was removed and transferred to a 96-well plate and processed according to the manufacturer's instructions.

### Western Blotting

Cells were incubated in either anchorage dependent or independent conditions for 48h. In another experiment, cancer cells were exposed to various concentrations of PL for 48h. Following these incubations, cells were collected, lysed and approximately 30-80 μg of protein was separated by sodium dodecyl sulfate (SDS) gel electrophoresis followed by immunoblotting as described previously[49].

### STAT3 transient transfection

STAT3 was either over-expressed using a plasmid or knocked down using shRNA as described previously by us [28]. Briefly, 0.3 × 10^6^ cells were plated in OPTI-MEM without antibiotics and transfected with scrambled shRNA, shRNA (STAT3 knock-down) or plasmid overexpressing STAT3 (STAT3 +/+). Complexes were prepared by incubating 2μg DNA with 6 μl Lipofectamine 2000 transfection reagent in 200 μl OPTI-MEM media without serum or antibiotic for 20 minutes. These complexes were then added to the cells. Six hours after transfection, complexes were replaced with fresh medium. After 24 hours of transfection, cells were processed for anoikis assay or western blotting as described above.

### Induction of STAT3 by IL-6

Activation of STAT3 (Y705) was performed as explained previously by us [28, 48]. Anchorage-independent cells were treated with 10ng/mL IL-6. One hour after IL-6 treatment, one group of cells was treated with 5μM PL for 48 hours. Following the treatment, cells were either processed for anoikis assay or western blotting.

### Generation of stable STAT3 knockout cell lines

STAT3 was knocked down stably in SK-MEL-28, SK-MEL-2, SK-MEL-5 and MeWo cells using STAT3 shRNA according to previously described method by us [48]. Colonies were selected using G418 as the selection marker. Selected colonies were then maintained with 2mg/mL of G418 till 8 passages. The transfected cells were then used for anoikis experiment, cell migration assay or *in vivo* experiments.

### Immunofluorescence

SK-MEL-28 cells were incubated with or without anchorage for 48h. Adherent and suspension cells were immunostained with anti-p-STAT3 (Y705) antibody as described previously by us [50].

### *In vivo* anoikis xenograft experiment

*In vivo* xenograft experiments were performed as described previously, with slight modifications[44]. Male SCID/NSG mice (5-7 weeks old) were obtained from TTUHSC Breeding Facility (Lubbock, TX) and maintained under special pathogen free conditions. The use of SCID/NSG mice was approved by the Institutional Animal Care and use Committee (IACUC) and the experiments were performed in strict compliance with the regulations. SK-MEL-28 cells were treated with 5 μM STAT3 inhibitor (PL) for 48h under anchorage-independent conditions. On the other hand, SK-MEL-28 STAT3 knock-down cells without treatment were incubated under anchorage-independent conditions for 48h. Viable cells were counted by trypan blue assay and 5×10^6^ cells resuspended in 1:1 Dulbecco Phosphate Buffered Saline (DPBS):Matrigel (BD Biosciences, Houston, TX) were injected subcutaneously in both flanks of the mice (3 groups; n=9 mice per group). Tumor volume was measured using vernier calipers twice a week and the tumor volume was calculated using the formula described by us previously[51, 52].

### *In vivo* anoikis metastasis experiment

Male SCID/NSG mice (5-7 week old) were used for this experiment. SK-MEL-28 wild type cells were treated with STAT3 inhibitor (PL) under anchorage-independent conditions for 48 h whereas SK-MEL-28 STAT3 knock-down cells were incubated in anchorage-independent conditions for 48h without any treatment. Untreated SK-MEL-28 cells incubated under anchorage-independent conditions were used as control. Viable cells were counted by trypan blue dye exclusion assay. About 0.2×10^6^ viable cells re-suspended in PBS were injected intravenously through the lateral tail vein (3 groups; n=10 mice per group). The experiment was continued till the mice became ill due to metastatic burden after which they were sacrificed, and lungs and liver were extracted for the analysis of metastatic tumor nodules by histopathology.

### Hematoxylin and Eosin (H & E) staining

H & E staining was performed on lungs and liver of the mice to detect the presence of the metastatic nodules, according to the previously described procedure[28]. Tissues extracted from mice were fixed in 4% formalin, dehydrated in series of solvents and embedded in paraffin. The sections with 5 μm thickness were prepared giving a gap of 100 μm using a microtome. Tissue sections were stained using H & E and imaged under the microscope (Olympus America Inc, Central Valley, PA).

### Statistical Analysis

All the statistical calculations were performed using Prism 6.0 (GraphPad Software Inc., San Diego, CA). The data was represented as mean ± S.D. or S.E.M. Student's t-test was used for comparison of two groups. For experiments involving more than two groups, ANOVA followed by Tukey's post hoc multiple comparison test was used. All the statistical tests were two sided. Differences were considered statistically significant when p value was < 0.05.
